# High Problematic Screen Exposure among Children Aged 2-5 Years Visiting the Department of Pediatrics of a Tertiary Care Centre

**DOI:** 10.31729/jnma.8364

**Published:** 2023-12-31

**Authors:** Henish Shakya, Sharda Acharya, Shikhar Pradhan, Divya KC

**Affiliations:** 1 Department of Pediatrics, KIST Medical College and Teaching Hospital, Mahalaxmi, Lalitpur, Nepal

**Keywords:** *children*, *preschool*, *screen time*

## Abstract

**Introduction:**

The effects of problematic screen exposure in the early years have adverse effects on cognition, reasoning, executive, and social skills, and physical health. The study aimed to assess the prevalence of problematic screen exposure in children between 2 to 5 years of age visiting the Department of Paediatrics of a tertiary care centre.

**Methods:**

A descriptive cross-sectional study was conducted among caregivers of children aged 2 to 5 years of age in a tertiary care centre from 16 July 2023 to 30 September 2023 after an ethical clearance from the Institutional Review Committee. This study excluded children with chronic disease and behavioural disorders. The problematic screen exposure was assessed using the Problematic Screen Exposure Score. A convenience sampling method was used. The point estimate was calculated at a 95% Confidence Interval.

**Results:**

Among 310 respondents, 216 (69.67%) (64.55-74.79, 95% Confidence Interval) children had a high problematic screen exposure score. The mean age of children was 3.46±1.033 with 89 (41.20%) children having their first exposure before 12 months of age, 131 (60.64%) exceeding daily usage of >2 hr, and 198 (91.66%) children viewing age-inappropriate media content.

**Conclusions:**

The prevalence of high problematic screen exposure was comparable to that of the prevalence found in studies done in similar settings.

## INTRODUCTION

Children are the fastest-growing users of digital media as a result of evolving technologies, increased family use of devices, and easy access. Screen time is the time spent using a device with a screen, such as a TV, computer, smartphone, tablet, or game console. The American Academy of Pediatrics recommends restricting screen time for children between the ages of 2 and 5 to one hour per day and completely avoiding screen except video conferencing for those younger than 2 years.^1^ The Indian Academy of Pediatrics has also adopted similar guidelines.^2^

Extended screen use in children has been associated with many detrimental effects on health and development, including obesity, behavioural issues, decreased quality of sleep, emotion management, learning difficulties, speech delays, and problems in forming social connections.^3,4^ The impact is greater in preschoolers, as these are formative years where habits are established more easily and usually continue into adulthood.^5^

The study aimed to find the prevalence of problematic screen exposure in children between 2 to 5 years of age visiting the Department of Paediatrics of a tertiary care centre.

## METHODS

This is a descriptive cross-sectional study conducted in KIST Medical College and Teaching Hospital, Imadol, Lalitpur, Nepal from 16 July to 30 September 2023. Ethical approval was obtained from the Institutional Review Committee of the same institute (Reference number: 2079/080/129). The study included primary caregivers of children aged 2 to 5 years who visited the outpatient Department of Pediatrics and who were admitted to the Paediatric Ward. Children with chronic illnesses and behavioural disorders were excluded. A convenience sampling method was used. The sample size was calculated using the following formula:


$$\begin{array}{ll} \text{n} & ={\text{Z}}^{2}\times \frac{\text{p}\times \text{q}}{{\text{e}}^{\text{2}}}\\ & =1.96^{2}\times \frac{0.50\times 0.50}{0.06^{2}}\\ & =267 \end{array}$$

Where,

n = minimum required sample size
Z = 1.96 at 95% Confidence Interval (CI)
p = prevalence taken as 50% for maximum sample size calculation
q = 1-p
e = margin of error, 6%

The calculated minimum required sample size was 267. However, a total of 310 sample size was taken in our study.

A pre-structured questionnaire was used. An informed consent was taken, and the questionnaire was filled out by the co-investigators. The first part of the survey included the demographics and the child's characteristics. The second part of the survey was designed based on the "Seven-in-Seven Screen Exposure Questionnaire" conducted to evaluate problematic screen exposure. All screen exposure characteristics were asked regarding the last month. The problematic screen exposure (PSE) Score was then calculated (range 0-13) based on the questionnaire. The screen time was calculated as screen time (weekdays) x 5 + screen time (weekends) × 2/7. The screen content was asked as an open-ended question. Age-appropriate and educational content was labelled as high-quality viewing, while fast-paced programs with violent and age-appropriate content were labelled as inappropriate content. Total scores were classified as low (<7) and high (>7) with higher scores indicating more problematic screen exposure.^6^

Data were entered and analysed by using IBM SPSS statistics version 21.0. The point estimate was calculated at a 95% CI.

## RESULTS

Among 310 respondents, 216 (69.67%) (64.55-74.79, 95% CI) children had a high problematic screen exposure. The mean age of children was 3.46±1.033 years. The primary caretaker were parents of 156 (72.22%) children (Table 1).

**Table 1:** Demographic characteristics (n= 216).

Family characteristics	n (%)
**Primary caretaker**	
Parents	156 (72.22)
Grandparents	47 (21.75)
Nanny	10 (4.62)
Daycare	3 (1.38)
**Family type**	
Nuclear	107 (49.53)
Joint/Extended	105 (48.61)
Single parent	4 (1.85)
**Paternal age**	
< 30 years	78 (36.11)
≥ 30 years	137 (63.42)
Expired	1 (0.46)
**Maternal age**	
<30 years	83 (38.42)
≥30 years	132 (61.11)
Expired	1 (0.46)
**Maternal occupation**	
Employed	90 (41.66)
Homemaker	115 (53.24)
Expired	1(0.46)

A total of 120 (55.55%) were males (Table 2).

**Table 2:** Characteristics of children (n= 216).

Child characteristics	n (%)
**Age of child (years)**	
2	42 (19.44)
3	79 (36.57)
4	49 (22.68)
5	46 (21.29)
**Gender**	
Male	120 (55.55)
Female	96 (44.44)
**Birth order**	
1	131 (60.64)
2	77 (35.64)
3	8 (3.7)
**Siblings**	
0	83 (38.42)
1	111 (51.38)
2	21 (9.7)
3	1 (0.46)

In the screen exposure characteristics, 89 (41.20%) children had their first screen exposure before 12 months of age. There was a preponderance of use of screen during mealtime in 190 (87.96%) chidren. The daily usage of media devices was >2 hr in 131 (60.64%). 149 (68.98%) of caretakers responded that they monitored the media content that their children were watching. 198 (91.66%) of children were viewing age-inappropriate media content (Table 3).

**Table 3:** Screen characteristics (n= 216).

Age of first screen exposure (months)	
≥24	8 (3.70)
18-23	37 (17.12)
12-17	82 (37.96)
<12	89 (41.20)
Use 1 hr before bedtime	146 (67.59)
Use during mealtime	190 (87.96)
Use within 1 hour after waking	93 (43.05)
**Daily Usage**	
< 1 hr	5 (2.31)
1-2 hr	80 (37.03)
>2 hr	131 (60.64)
**Setting Screen Limit**	
No limit	62 (28.70)
Setting and obeying	111 (51.38)
Setting but not obeying	43 (19.90)
**Monitoring media content**	
No	67 (31.01)
Yes	149 (68.98)
**Media content**	
Age appropriate	18 (8.33)
1 inappropriate	137 (63.42)
≥ 2 inappropriate	61 (28.24)
**Co-watching with children**	
Always	1 (0.46)
Sometimes	99 (45.83)
Rarely	116 (53.70)

## DISCUSSION

With the advent of the digital age, screen media devices have become an inseparable part of daily life. Early and problematic exposure to screen media devices has also risen alarmingly in younger children. High screen time in children has been known to be associated with sedentary lifestyles leading to obesity, mood swings, and behavioural and sleep disorders, and has a negative effect on the cognitive development of the child.^3,4^ Our study showed a prevalence of problematic screen exposure based on the PSE score to be 69.7%. Similar studies on screen time in children done in Nepal showed a prevalence of 55.2% in children aged 3 to 10 years of age^9^ and 47.4 % in 5-9 years old.^10^ These studies focused on school-aged children while preschoolers were not the predominant study groups. A review article in India showed a prevalence of 59.5 % in children under 5 years of age.^7^ In a study in India done in 2022, >80% of children aged 2 to 5 years of age exceeded the recommended screen limit. The high prevalence was attributed to the COVID-19.^11^ A metaanalysis of 44 studies in 2022, revealed that 64.4% of children aged 2 to 5 years exceeded the screen limit guideline.^8^ The first five years are the formative years and the health routines and habits established early tend to impact the adult life.^5^ The risk associated with excessive screen time during these initial years thus poses a significant risk and more studies are warranted to assess the real burden of the problem.

The mean age of screen exposure was 3.46±1.033 years in our study with 41.20% of children having their first exposure to screen before 12 months of age whilst 37.96% had their first exposure between 12 to 17 months of age. The daily usage of screen media exceeded 2 hours in 60.64% of children. In a study done in India, 53% of children had screen exposure before 2 years of age.^11^ In a study in Virginia, the daily screen time ranged from 0.1 to 5 hours among under-fives with maximum duration reported in 3-5 years of age.^12^ According to a report by the CDC in 2020, 47.5% of children aged 2-5 years in the US spent more than 2 hours of screen time per weekday.^13^ AAP does not recommend the use of screen media other than video chatting for children under 18 months of age.^1^ Early exposure to screen is known to harm the developing brain leading to addictive behavior later in life and impairment of language, cognition, and executive skills.^1,3,4^

In our study, 87.96% of children used screen media during mealtime, and 67.59% used it 1 hour before bedtime. A study in India showed about 77% of parents took the help of a screen to feed their children.^11^ A study done in Toronto showed at least 59% of children consumed at least 1 meal while watching the screen. They also showed a significant association of higher screen time with its use during mealtime.^14^ It has been associated with impaired satiety, decreased family interaction, and risk of obesity.^5^ In a systematic review published in 2021, a strong association has been demonstrated between the use of screens before bedtime with sleep impairment.^4^

In the evaluation of screen media parenting practices, our study showed that although 68.98% of caretakers responded that they monitored the screen content, only 46.29% were co-viewing with their children and 91.66% of the children were exposed to age-inappropriate media content. Different studies done in India^11^ and Pokhara^15^ showed that there was a lacuna in parental supervision in setting a time limit and content restriction. AAP recommends parental supervision in the form of active supervision, co-viewing, restricting time spent on screen, and restricting the content.^1^ This is to mitigate screen time because when children watch educational, age-appropriate content with an engaged adult, screen time can be a positive learning experience.^1,5^

There are certain limitations to our study. As it is a hospital-based study catering to a particular demographic and age group, it does not reflect the community prevalence. It also does not consider a selfreporting bias that could occur if participating mothers are aware of screen time appropriateness. This study thus highlights the need to garner awareness of excessive screen time in small children as an emerging public health problem and its long-term impact.

## CONCLUSIONS

The prevalence of problematic screen exposure was higher than in other studies done in similar settings.

Policymakers should focus on formulating guidelines on permissible and quality screen time in children and ensure awareness among the general public regarding the hazards of excessive screen exposure.
